# Dissection of the Beta-Globin Replication-Initiation Region Reveals Specific Requirements for Replicator Elements during Gene Amplification

**DOI:** 10.1371/journal.pone.0077350

**Published:** 2013-10-04

**Authors:** Naoya Okada, Noriaki Shimizu

**Affiliations:** Graduate School of Biosphere Science, Hiroshima University, Higashi-hiroshima, Hiroshima, Japan; CCR, National Cancer Institute, NIH, United States of America

## Abstract

Gene amplification plays a pivotal role in malignant transformation of human cells. A plasmid with both a mammalian replication-initiation region (IR)/origin/replicator and a nuclear matrix-attachment region (MAR) is spontaneously amplified in transfected cells by a mechanism that involves amplification at the extrachromosomal site, followed by amplification at the chromosomal arm, ultimately generating a long homogeneously staining region (HSR). Several observations suggest that replication initiation from IR sequences might mediate amplification. To test this idea, we previously dissected c-myc and *DHFR* IRs to identify the minimum sequence required to support amplification. In this study, we applied an improved analysis that discriminates between two amplification steps to the ß-*globin* RepP IR, which contains separate elements already known to be essential for initiation on the chromosome arm. The IR sequence was required at least for the extrachromosomal amplification step. In addition to the vector-encoded MAR, amplification also required an AT-rich region and a MAR-like element, consistent with the results regarding replicator activity on the chromosome. However, amplification did not require the AG-rich tract necessary for replicator activity, but instead required a novel sequence containing another AG-rich tract. The differential sequence requirement might be a consequence of extrachromosomal replication.

## Introduction

Amplification of oncogenes or drug-resistance genes, resulting in overexpression of the encoded proteins, plays a pivotal role in malignant transformation of mammalian cells. Highly amplified genes frequently reside on extrachromosomal double minutes (DMs) or chromosomal homogeneously staining regions (HSRs) of various lengths. We previously found that a plasmid with a mammalian replication-initiation region (IR)/origin/replicator and a nuclear matrix-attachment region (MAR) spontaneously initiates gene amplification and generates DMs or HSR *de novo* in transfected cells [1,2]. The MAR sequence mediates the attachment to the nuclear matrix, which is required for the DNA replication initiation (for a recent review, see 3). We employed this “IR/MAR gene amplification” method to explore the intranuclear folding and replication of large HSR [4], the intracellular behavior of DMs [5], the specific binding of tramscription factor to the target sequence [6], and the transcription from either DMs [7] or HSR [8]. We also employed the novel amplification method to efficiently produce recombinant protein [9-12], and to investigate the mechanisms of gene amplification [13,14].

**Figure 1:**
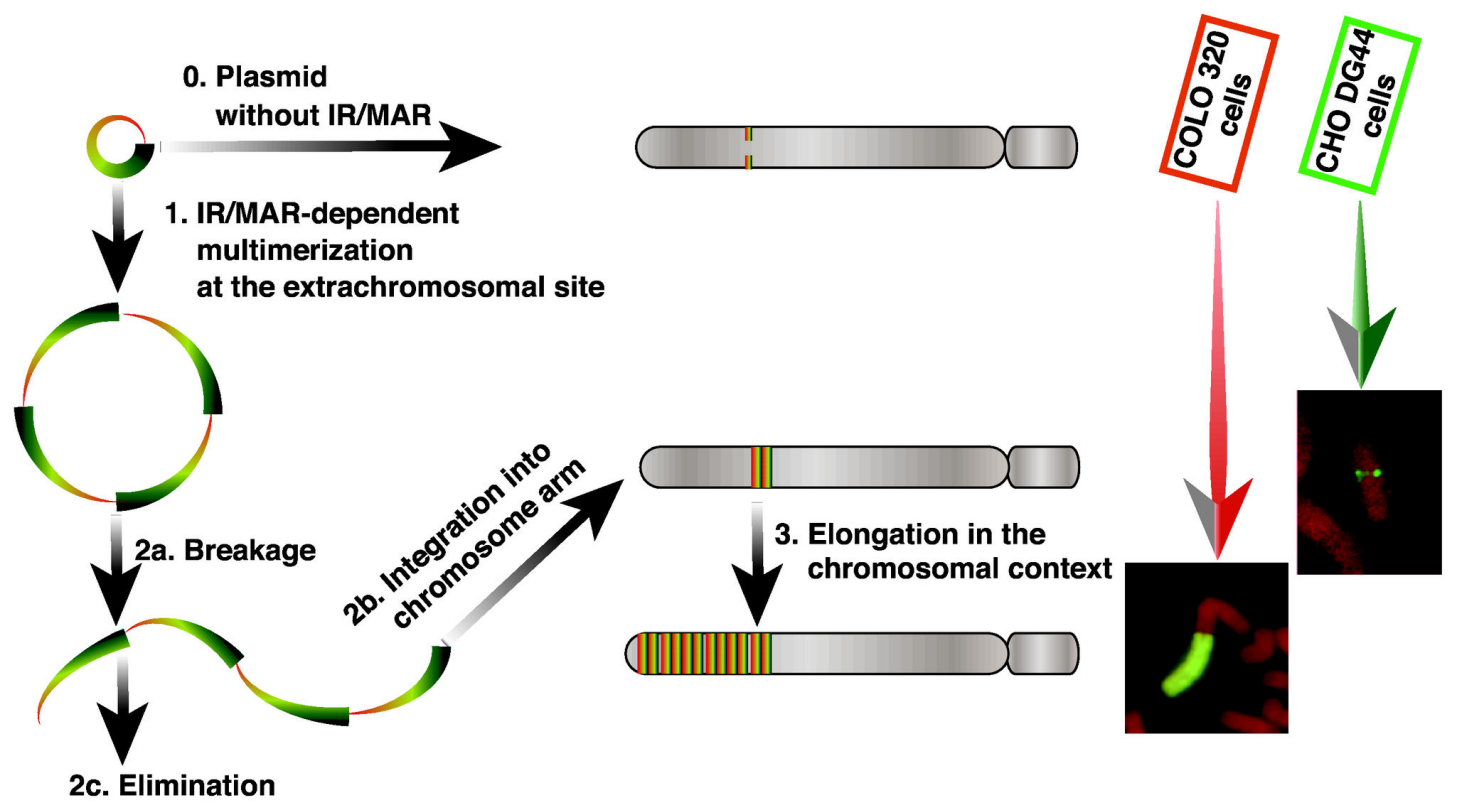
Amplification of the IR/MAR plasmid in transfected cells. After transfection, a conventional plasmid without an IR/MAR is integrated into the chromosome arm at low copy number in a stable transfectant. Such a sequence is barely detectable by FISH (noted as “0”). By contrast, an IR/MAR-bearing plasmid is amplified at an extrachromosomal location as a large circular molecule of tandem plasmid repeats (step 1; [2]). If such a molecule suffers from a DNA break (step 2a), it is eliminated from cells (step 2c; [5]) or integrated to a chromosome arm, generating a small HSR that is visible by FISH as paired dots or a line (step 2b). There, the plasmid repeat is elongated to generate a large HSR (step 3; [13,14]). These three steps occur spontaneously in human COLO 320DM cells, whereas step 3 does not occur spontaneously in CHO DG44 cells [11].

The IR/MAR plasmid is initially multimerized, and is maintained in transfected cells as an extrachromosomal circular molecule in which the plasmid sequence is arranged as a direct repeat (Figure 1; step 1 [2]). Fluorescence in situ hybridization (FISH) using a plasmid probe can detect multiple extrachromosomal molecules of various sizes in metaphase spreads from transfectants [1,2]. If such a circular molecule suffers from a double-strand break, it is either eliminated from the cell [5] or integrated into a chromosome arm to generate a small HSR (Figure 1; step 2). In human colorectal carcinoma COLO 320 cells, such chromosomal tandem plasmid repeats spontaneously elongate to generate long HSRs (Figure 1; step 3), through either the breakage-fusion-bridge (BFB) cycle [2,13] or break-induced replication [14]. Spontaneous HSR elongation was active in COLO 320 cells, whereas it was inactive in hamster CHO-DG44 cells; therefore, HSRs remained short in the latter cell type (Figure 1 [11]).

Both the IR and MAR sequences appeared to be required for gene amplification. In particular, some information within the IR sequence is necessary for HSR generation, because an unrelated sequence of similar length did not support the process [18]. Furthermore, the Ig κ intronic AR1 sequence [15], which exhibits strong *in vitro* MAR activity, elevated the level of HSR generation in combination with *DHFR* IR, and was indispensable for HSR generation in combination with c-myc IR. Consistent with these findings, the *DHFR* IR contains sequence with *in vitro* MAR activity [1]. Furthermore, blockage of progression of the replication fork or transcription machinery by an orientation-dependent replication fork barrier (RFB) sequence or the polyA addition sequence prevents generation of HSRs [2]. These results suggested that the HSRs are generated most efficiently if the plasmid is oriented such that the predicted replication fork from the IR sequence collides at the MAR with transcription machinery coming from the promoter [2]. Such collisions might produce DNA strand breakage [16]; DNA breakage and the malfunctioning of cell-cycle checkpoints are frequently associated with gene amplification [17]. Therefore, we previously developed a “plasmid-stability assay” that detects the HSR-generation activity of given DNA sequence [18] by using a plasmid vector (pTV-MCS). The vector has an arrangement in which if the replication initiate from the inserted test sequence, it collide with the transcription machinery from the promoter at the MAR. Using this assay, we dissected the *DHFR* IR (4.6 kbp) and the c-myc IR (2.4 kbp), both of which are early replicating origins [19], in order to identify the determinants of HSR generation in COLO 320 cells [18]. We successfully determined short sequences, 0.8 kbp for the c-myc IR and 1.7 kbp for the *DHFR* IR, which induce HSR generation as efficiently as the full IR sequences. Importantly, these short sequences contained the sequence elements required to support the replication initiation. Therefore, replication initiation from an IR sequence seems to be essential for gene amplification, but it is not clear which stage of gene amplification (Figure 1) requires initiation.

**Figure 2:**
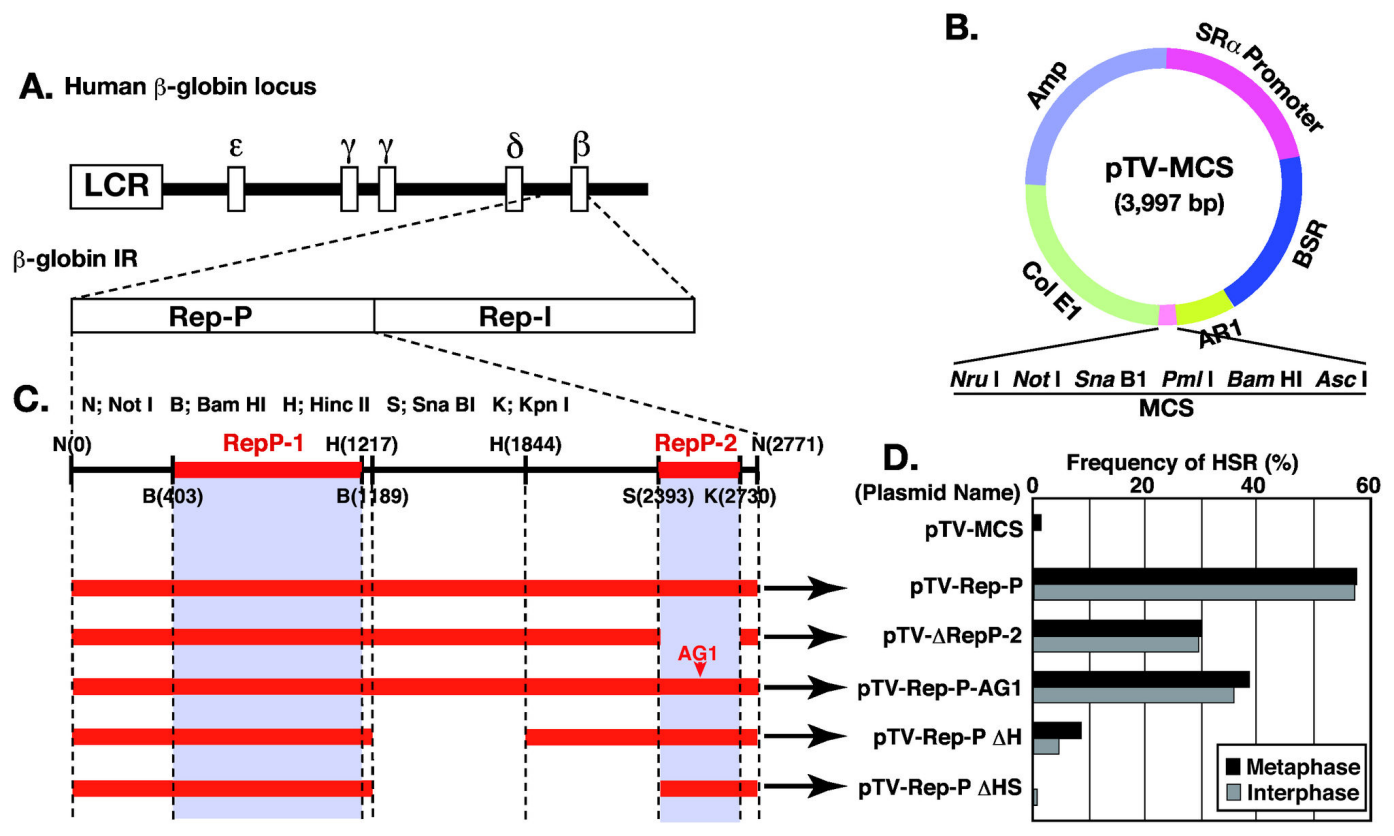
Dissection of the ß-globin replicator using a plasmid-stability assay **(1)**. The ß-globin locus and the positions of two reported replicators, Rep-P and Rep-I [20], are drawn in A. We inserted wild type Rep-P as well as fragments bearing four deletions or an AG1 point mutation (orange bars in C) into the *Not*I site of the vector plasmid pTV-MCS (B). In panel C, RepP-1 and RepP-2 regions are indicated as gray rectangles. The plasmids were transfected into COLO 320DM cells, and polyclonal transfectants were selected in blasticidine for about 1 month. Metaphase spreads from these cells were analyzed by FISH using a pTV-MCS plasmid-derived probe. The frequencies of cells bearing HSRs of plasmid repeats were scored in both metaphase and the interphase cells, and the data are plotted in panel E.

In this study, we dissected the IR sequence from the ß-globin locus, which replicates at flexible times depending on gene expression. The replicator activity of this region has been thoroughly studied in the context of replication initiation at an ectopic chromosomal locus [20,21]. Those studies demonstrated that the ß-globin IR consists of two independent replicators, Rep-P and Rep-I (Figure 2A). In Rep-P, replicator activity is derived from the combination of RepP-1 and RepP-2. RepP-2 contains an AG-rich sequence, a two-base mutation of which abolishes replicator activity [22]. We anticipated that a comparison between those results and the results of our HSR-generation assay might provide insight into both gene amplification and replication initiation. We performed the dissections in two cell lines, COLO 320DM cells and CHO-DG44 cells. Our previous study using these cell lines [11] suggested that the IR/MAR plasmid spontaneously undergoes extrachromosomal multimerization (Figure 1, step 1) in both cell lines, however, the elongation in a chromosomal context (Figure 1, step 3) does not proceed in CHO-DG44 cells (Figure 1). Therefore, our data might provide insight to the role of IR sequences in gene amplification.

## Materials and Methods

### Plasmid Construction

pTV-MCS was constructed by inserting a double-stranded synthetic oligonucleotide containing the recognition sequences of *Asc*I, *Bam*HI, *Pml*I, *Sna*BI, *Not*I, and *Nru*I (in that order) between the *Asc*I and *Nru*I sites, separated by 21 bases, of plasmid pTV [18].

Plasmid pJS_Rep-P, pJS_∆Rep-P, and the pJS_Rep-P AG1 mutant, which carries a two-base mutation in the AG-rich tract of the RepP-2 region, were kind gifts from Mirit I. Aladjem (NCI, NIH). pTV-Rep-P, pTV-∆RepP-2, and pTV-Rep-P-mutant were constructed by inserting the *Not*I fragments of pJS_Rep-P, pJS_∆RepP-2, and pJS_Rep-P mutant into the *Not*I site of pTV-MCS. pTV-Rep-P∆H and pTV-Rep-P∆HS were constructed by digesting pTV-Rep-P with *Hinc*II or *Hinc*II/*Sna*BI, respectively, followed by re-ligation. pTV-RepP-1 was constructed by inserting the RepP1 fragment obtained by *Bam*HI digestion of pTV-Rep-P into the *Bam*HI site of pTV-MCS.

pG1–pG16 and pG20–pG24 contained various portions of the Rep-P replicator at the *Not*I site of pTV-MCS. These plasmids were constructed by the following method. Primer3Plus (http://www.bioinformatics.nl/cgi-bin/primer3plus/primer3plus.cgi/) and the In-Fusion Primer Design tool (http://bioinfo.clontech.com/infusion/convertPcrPrimersInit. do) were used to design primers for amplification of the target sequence inside the Rep-P replicator. These primers have 15-base homology with the sequence flanking both ends of *Not*I-linearized pTV-MCS. The target DNA fragment was PCR amplified using these primers, with pTV-RepP DNA as the template. The PCR product was digested with *Not*I and ligated into *Not*I-linearized pTV-MCS. pTV-MCS∆AR1 was constructed by removing the *DHFR* IR from p∆BN [2] by *Not*I digestion and re-ligation. The resulting plasmid has an identical structure to pTV-MCS, minus the AR1 sequence, except that the latter plasmid has a short multiple cloning site (MCS) sequence. pTV-Rep-P∆AR1 and pG5∆AR1 were constructed by inserting the Rep-P replicator or the G5 sequence, respectively, into the *Not*I site of pTV-MCS∆AR1. In that case, Rep-P replicator was obtained from the *Not*I digestion of pRepP, and the G5 sequence was obtained by PCR.

All plasmid DNA was cloned in *E. coli* DH5αlpha, grown, and purified using the PureLink HiPure Plasmid Midiprep kit (Life Technologies, Inc.).

### The plasmid-stability assay

The plasmid-stability assay was performed as described [18]; namely, we inserted the test sequence into the *Not*I site of pTV-MCS (Figure 2B). The plasmid has an arrangement in which if the replication initiate from the inserted test sequence, it collides with the transcription machinery from the promoter of blasticidine-resistant gene. Such collision occurs around the AR1 MAR, and generates the long HSR. The resulting plasmid was cloned in *E. coli*, and plasmid DNA was purified as described above.

Human colorectal carcinoma COLO 320DM cells [23] and hamster CHO-DG44 cells [11] were obtained and cultured as described previously. Transfection of plasmid DNA was performed using the GenePORTER 2 Transfection Reagent (Genlantis, Inc.) in the case of COLO 320DM cells, or Lipofectamine 2000 (Life technologies, Inc.) in the case of CHO-DG44 cells. Transfected cells were selected in the presence of 5 µg/ml (for COLO 320DM cells) or 2 µg/ml (for CHO-DG44 cells) blasticidine S hydrochloride for about 1 month. From the polyclonal transfectants at this time point, we prepared metaphase chromosome spreads and performed FISH using a digoxigenin-labeled probe prepared from pTV-MCS DNA. The procedure was as described in our previous paper [18]. To determine the frequency of HSR-bearing cells, we examined more than 60 metaphase cells and more than 600 interphase cells; detection of HSRs was more accurate in the metaphase cells, whereas we could examine larger numbers of interphase cells.

## Results

### Dissection of the ß-globin IR according to the ability to promote gene amplification in COLO 320 cells

**Figure 3:**
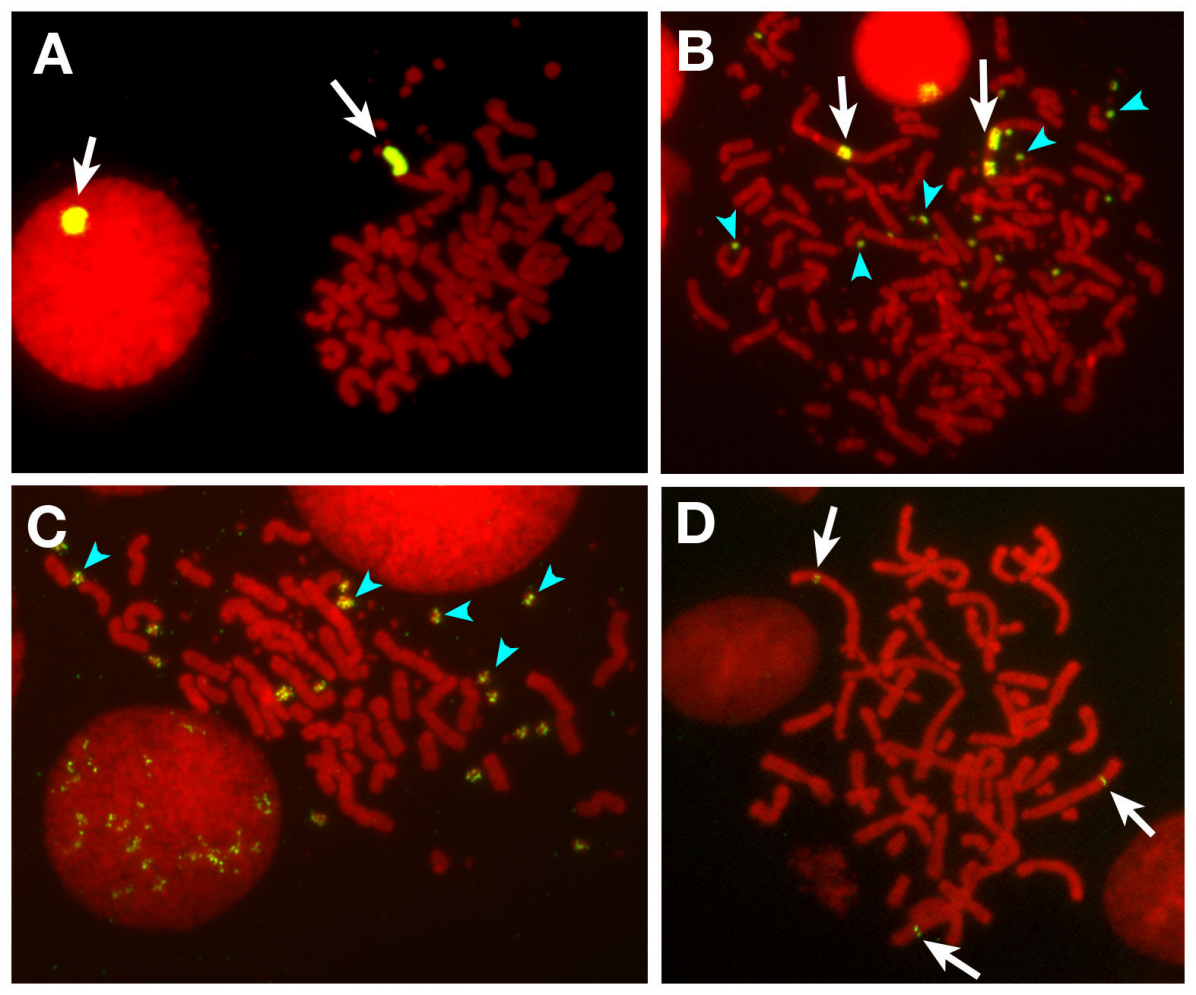
Detection of the amplified plasmid sequence by FISH. Representative images obtained in this study are shown. Plasmid pTV-Rep-P bearing full-length Rep-P sequence was transfected to COLO 320DM cells (A to C) or CHO-DG44 cells (D). Metaphase chromosome spreads were prepared from the stable transfectants, and the plasmid sequence was detected by FISH. Long HSR appeared in the metaphase cells (A and B) or a interphase cell (A). The DMs with plasmid sequence appear in the metaphase cells in panel B and C as well as an interphase cell in panel C. In CHO-DG44 cells, the same plasmid did not generate long HSR, instead it remained as the short HSRs that were arrowed (D), as explained in Figure 1.

The human ß-globin IR sequence is composed of two replicators, Rep-P and Rep-I (Figure 2A [20]). We dissected Rep-P using the plasmid-stability assay [18]. To this end, we cloned the test sequence at the *Not*I site of pTV-MCS (Figure 2B). The orientation of the resulting plasmid is such that promoter-driven transcription machinery might collide at a MAR (AR1) with the hypothetical replication fork from the test sequence. Such an orientation efficiently generates HSRs [2]. We transfected this plasmid into COLO 320DM cells, selected for stable transfectants, and analyzed them by FISH to detect HSRs composed of plasmid sequence. A plasmid containing the full-length Rep-P sequence (pTV-Rep-P) was efficiently amplified, and it generated DMs and HSR as assessed by FISH examination using probe prepared from pTV-MCS (Figure 3A to C). There were the metaphase cells with long HSR (Figure 3A), with multiple DMs (Figure 3C) and with both DMs and HSR (Figure 3B). It was consistent with our previous results that were obtained from the plasmid with *DHFR* IR or c-myc IR [1,2], and it was consistent with that the extrachromosomal DMs generate the chromosomal HSR. The HSR (Figure 3A) and the DMs (Figure 3C) could be identified also in the interphase nucleus. Because we previously found that the frequency of DMs fluctuate between experiments and that the frequency of HSR was quite reproducible [18], we counted the frequency of long HSR among both metaphase and interphase cells (Figure 2D). On the other hand, a plasmid without the inserted IR fragment (pTV-MCS) barely generated HSRs (Figure 2D). These results were consistently reproducible in subsequent experiments. Furthermore, we previously found that unrelated DNAs of similar size did not generate HSRs [18]. Thus, the generation of HSRs depended strictly on the sequence information within the IR.

For replicator activity at the ectopic chromosomal locus, both the RepP-1 and RepP-2 regions of Rep-P are required (Figure 2A [21]). In particular, the AG-rich sequence inside RepP-2 is required, as demonstrated by the observation that mutation within this sequence (AG1) abolished replicator activity [22]. Therefore, we tested a series of deletion mutants (∆RepP-2, ΔH, and ∆HS) and the AG1 mutant of RepP-2, using the plasmid-stability assay. Unexpectedly, HSR generation was not abolished by deletion of RepP-2, although the level of HSRs was decreased (Figure 2D). The effects of the point mutation in RepP2 were almost the same as those of the RepP2 deletion. Furthermore, although a previous analysis of replicator activity at the ectopic locus suggested that the sequence between RepP-1 and RepP-2 is not required [20], our plasmid-stability assay demonstrated that deletions in this region significantly (ΔH) or almost completely (∆HS) abolished HSR-generation activity (Figure 2D). Essentially similar results were obtained from two independent transfection experiments, and one of which is not shown.

**Figure 4:**
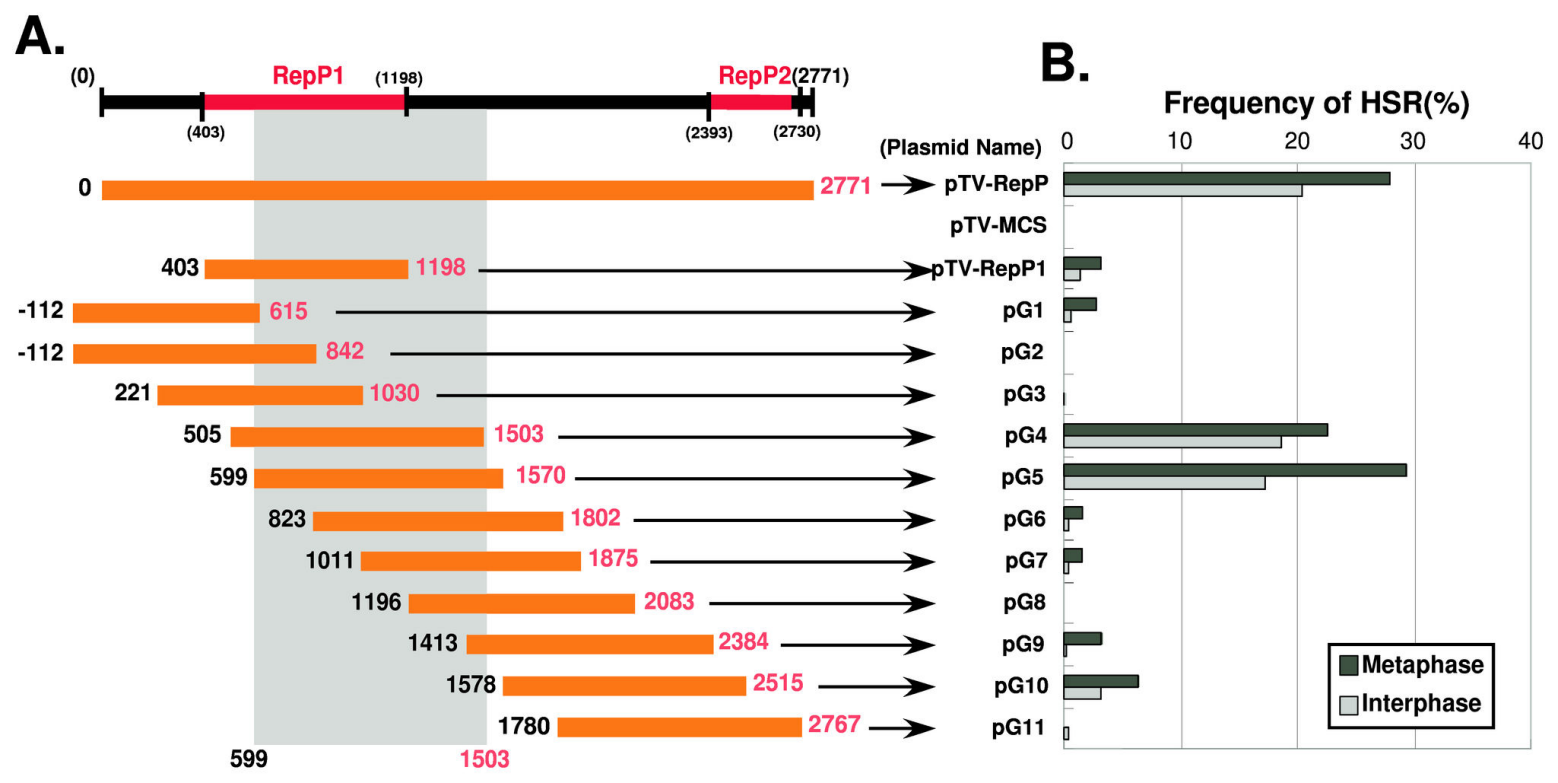
Dissection of ß-globin replicator using a plasmid-stability assay (2). The regions indicated by the orange bars in the left panel were subjected to the plasmid-stability assay in COLO 320DM cells, as in Figure 2. The results are shown in the right panel. The data suggest that the gray rectangular region drawn in the left panel has HSR-generation activity.

Next, we further systematically dissected the entire 2.7 kbp Rep-P replicator, in order to identify the minimal sequence that supports HSR generation. At the first stage, we screened a series of approximately 1 kbp fragments in sliding intervals of 100–250 bp (Figure 4). Again, the results suggested that RepP-2 is dispensable for HSR generation, whereas a portion of RepP-1 and its flanking region (599–1503) is required. Therefore, we next dissected the pG5 sequence, which exhibited the highest HSR-generation activity in this assay (as high as the intact Rep-P).

**Figure 5:**
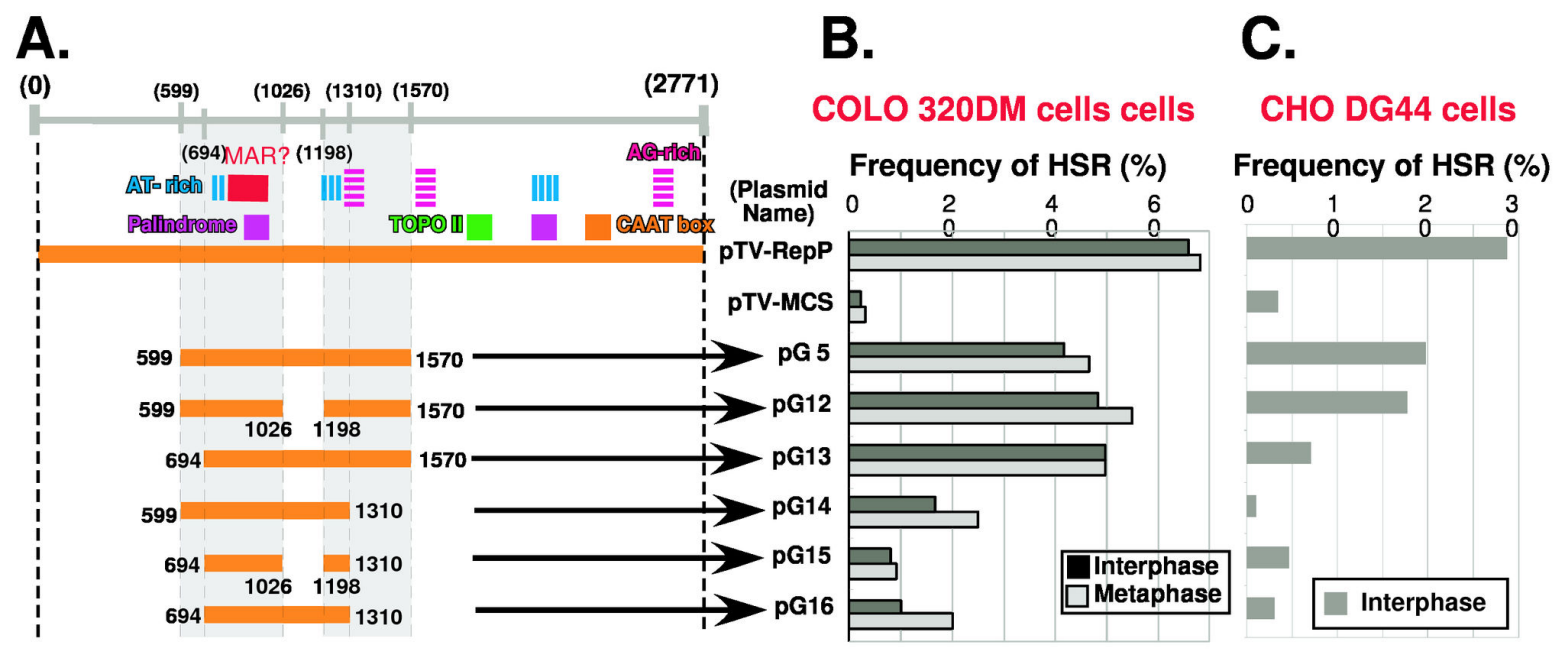
Dissection of ß-globin replicator using a plasmid stability assay **(3)**. The G5 fragment, which exhibited the highest HSR-generation activity in Figure 3, was further dissected. The positions of AT-rich, AG-rich, palindrome, topo II binding-site, and CAAT-box regions are indicated. “MAR?” indicates the position of a MAR-like element predicted by the MAR-Wiz program. The regions indicated by orange bars in panel A were subjected to the plasmid-stability assay as in Figure 2. In this case, we examined HSR generation in both COLO 320DM cells and CHO-DG44 cells, in which gene amplification progresses differently, as shown in Figure 1. The results from both cell lines are shown in panels B and C. The data suggest that the gray rectangular regions drawn in the left panel have HSR-generation activity.

Because pG5 contains two AT-rich regions and a sequence that is assigned a higher score by the MAR-Wiz program (i.e., a MAR-like element; see below), we specifically examined the effects of these sequences (Figure 5). The leftmost sequence flanking the first AT-rich region (599–694) is not required, because pG13 generated HSR as frequently as pG5. The sequence between the MAR-like element and the second AT-rich region (1026–1198) is also dispensable, because pG12 was as active as pG5. On the other hand, the right portion of the second AT-rich region (1310–1570) appears to be required, because pG14 to pG16 exhibited lower HSR-generation activity than pG5, pG12, and pG13.

**Figure 6:**
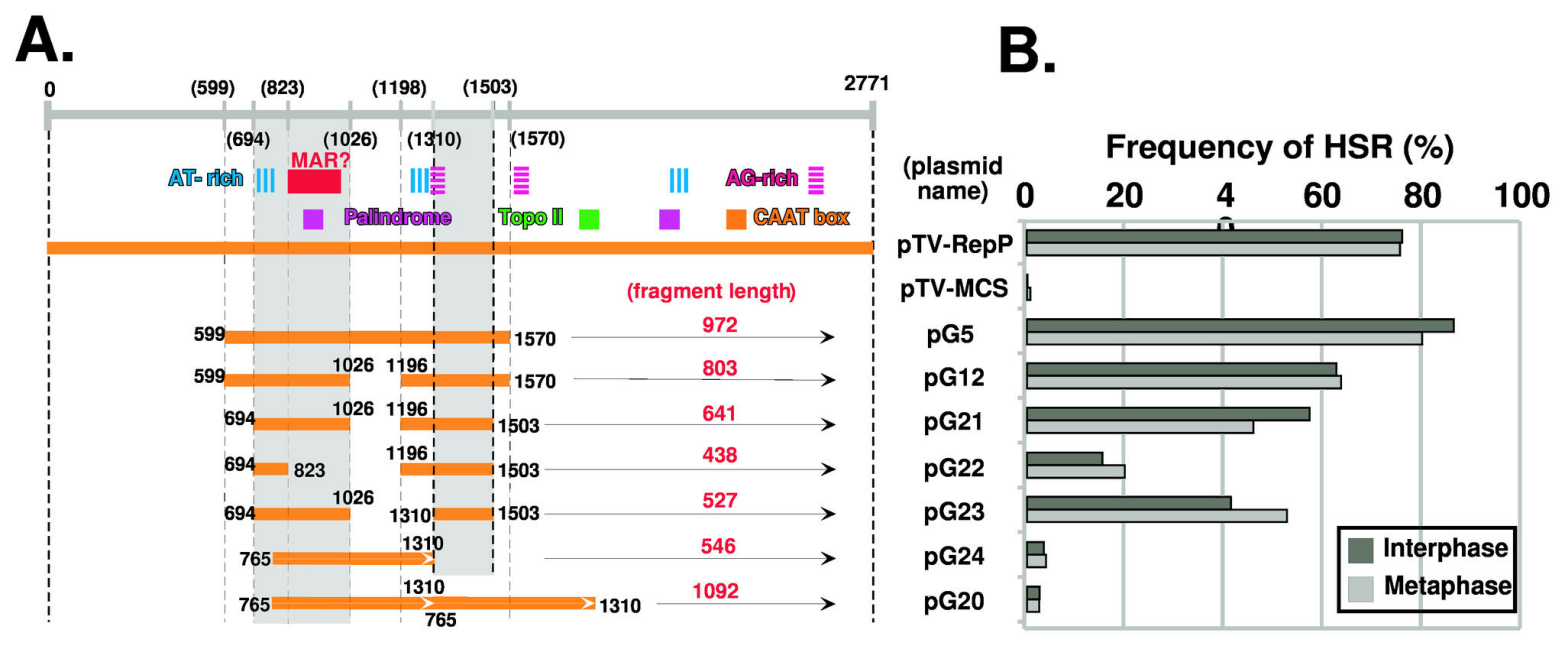
Dissection of the ß-globin replicator using a plasmid stability assay **(4)**. The regions indicated by orange bars in panel A were subjected to the plasmid-stability assay in COLO 320DM cells, as in Figure 2. pG20 contains a direct repeat of fragment G24 (765-1310). The results from both cell lines are shown in panel B. The data suggest that the gray rectangular regions drawn in the left panel have HSR-generation activity.

Based on the results from pG5, pG12, and pG13, we further dissected the region (Figure 6). The results again suggested that both the leftmost sequence until the first AT-rich region (599–694) and the rightmost sequence (1503–1570) are dispensable (compare pG21 to pG12). The MAR-like element is indispensable, because pG22 exhibited rather low HSR generation compared to pG21. The second AT-rich sequence is not needed, because pG23 HSR-generation activity was as high as that of pG21. These observations suggested that the presence of the first AT-rich sequence alone is sufficient for the HSR generation. On the other hand, the region from 1310–1503 is required, because it is contained within the necessary sequence from 1310–1570 (Figure 5, compare pG5 and pG14), whereas the region from 1503–1570 is dispensable (Figure 6, compare pG12 and 21). Consistent with this, neither pG24, which lacks the first AT-rich region and the asymmetric AG tract, nor its tandem dimer (pG20) generated significant levels of HSRs. Thus, pG23 was the shortest sequence that efficiently generated HSRs. This sequence contains the AT-rich tract (744–772; 28 bases, 92.8% AT), the MAR-like element (809–1009), and a third sequence (1310–1503) containing the AG-rich tract (1310–1346; 37 bases, 78.4% AG), which is a portion of a longer tract (1298–1346; 49 bases, 79.6% AG). Below, we will discuss these findings in relation to the published data.

### Gene amplification in CHO DG44 cells by a shortened ß-globin IR

Until this point, we had dissected the ß-globin IR according to HSR-generation ability in COLO 320DM cells. In these cells, the IR/MAR plasmid spontaneously undergoes both extrachromosomal multimerization (Figure 1, step 1) and elongation in a chromosomal context (Figure 1, step 3). We next examined gene amplification in CHO-DG44 cells, in which the IR/MAR plasmid undergoes only the extrachromosomal multimerization step, and is not elongated in a chromosomal context [11]; therefore, this plasmid generates only small HSRs. We transfected the same plasmids used for COLO 320DM cells in Figure 5B into CHO-DG44 cells; as before, the transfectants were selected and analyzed by FISH using a plasmid probe. The results revealed that the sequence responsible for HSR generation was quite similar between the cell lines (compare Figure 5B and C), suggesting that the IR sequence is, at least, required for the extrachromosomal multimerization step.

### Effect of MAR on gene amplification

**Figure 7:**
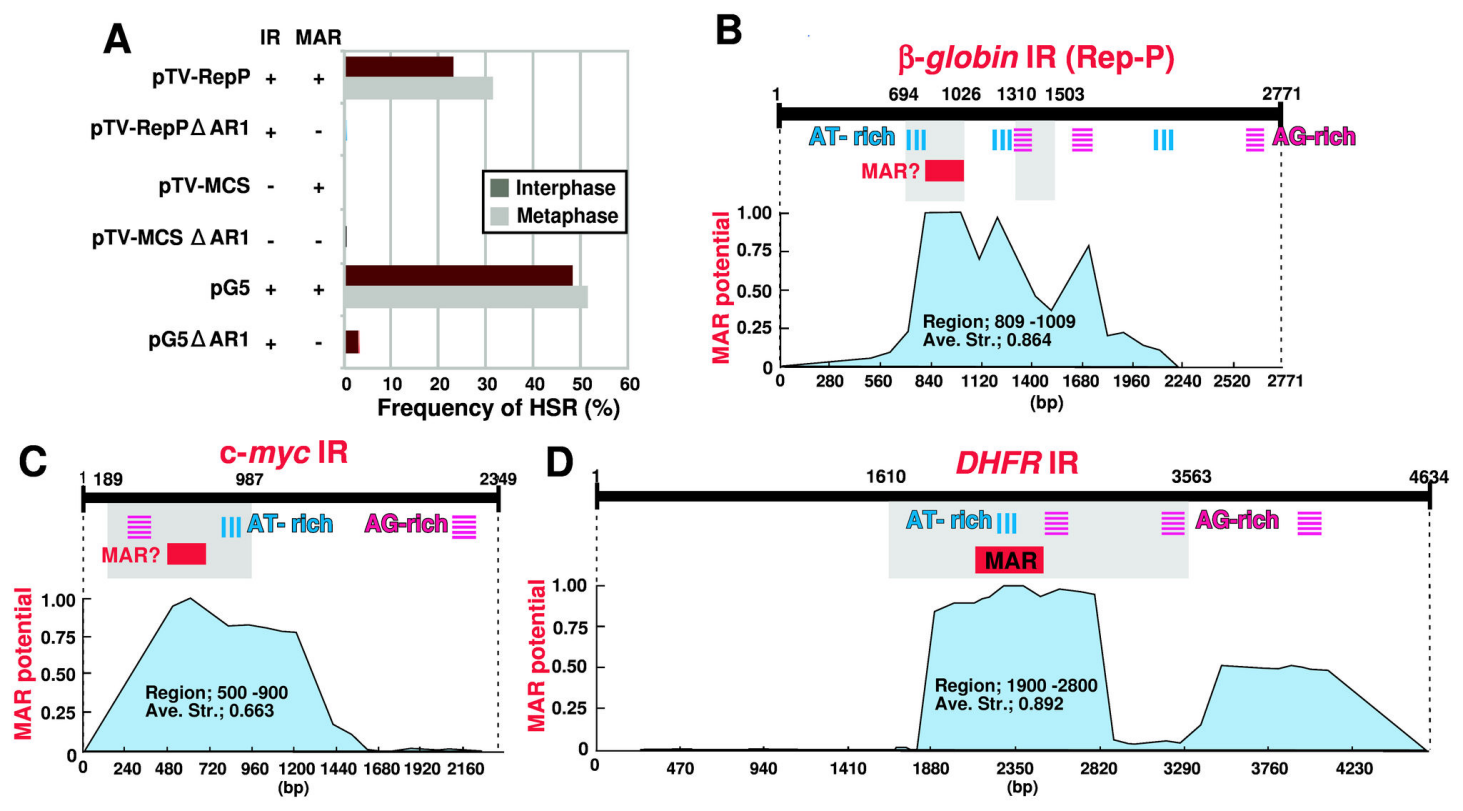
Requirement of MAR for HSR generation. A. To examine the effect of the AR1 MAR sequence of the vector plasmid, we removed it and constructed three new plasmids, pTV-Rep-P∆AR1, pTV-MCS∆AR1, and pG5∆AR1. We transfected plasmids with or without the AR1 MAR into COLO 320DM cells, selected transfectants, and analyzed them by FISH. Frequency of HSR is plotted in the graph. B–D. The IR sequences from ß-globin Rep-P (B), c-myc (C), and *DHFR* (D) were analyzed using MAR-Wiz program, and the results (blue graph area) are shown. In each graph, regions with HSR-generation activity revealed by our previous study (*DHFR* and c-myc; [18]) and this study (ß-globin) are shown as gray rectangle(s). AT-rich and AG-rich tracts are also indicated.

The experiments described above strongly suggested that the MAR-like element in Rep-P predicted by the MAR-Wiz program is required for the HSR generation. However, the plasmid pTV-MCS, which was used as a vector to clone the target IR fragment, already contains an exogenous MAR, i.e., the AR1 sequence that actually bound to the nuclear matrix in vitro [15]. Therefore, we examined whether the AR1 sequence in the vector might be dispensable for the generation of HSRs. To this end, we deleted the AR1 sequence and examined the effect on HSR generation (Figure 7A). The full-length Rep-P (pTV-Rep-P) and its shortened fragment (pG5) generated HSRs as frequently as before. On the contrary, the corresponding plasmid without AR1 (pTV-Rep-P ∆AR1 and pG5 ∆AR1) barely generated HSR, as did plasmids with (pTV-MCS) or without (pTV-MCS ∆AR1) AR1. This result suggested that in addition to the MAR-like element in the IR sequence, the AR1 MAR in the vector is also required.

## Discussion

We have dissected the ß-globin IR using our plasmid-stability assay, which measures HSR-generation activity. As described earlier, our previous studies provided several lines of evidence that the HSR-generation activity of a sequence might coincide with its replicator activity [2,18]. This study added further evidence that sequence information with the IR is required for amplification. Furthermore, the results suggest that the IR sequence is required at least for the extrachromosomal amplification/replication of the plasmid, because the same short sequence supported gene amplification in both COLO 320DM and CHO DG44 cells (Figure 5).

**Figure 8:**
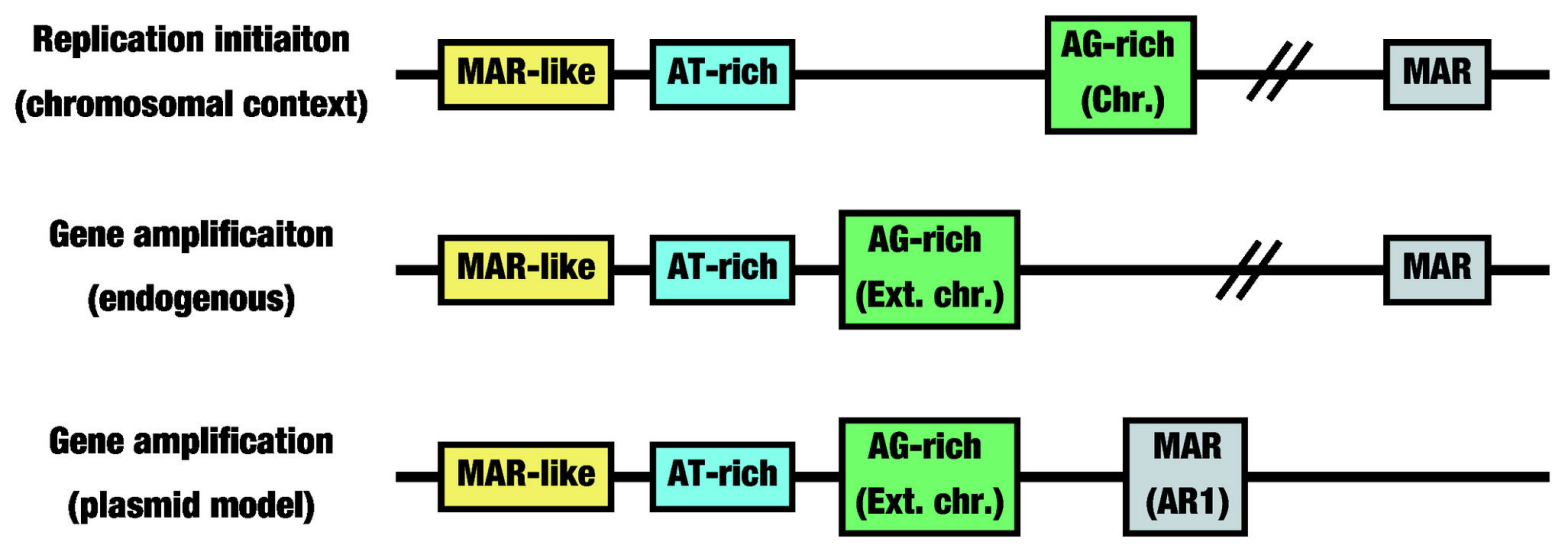
A model from this study. The replication initiation at the chromosomal context, the gene amplification from the endogenous chromosomal sequence and the gene amplification from the plasmid sequence require the same MAR-like element and the same AT-rich element, whereas the former one and the latter two events require similar but different AG-rich elements. The MAR elements is also required for these three events, and it is included in the plasmid (AR1) or it may be located at the chromosomal flanking region.

Our data indicate that, in addition to the vector coded MAR, three regions are required for gene amplification: the AT-rich tract (744–772), the MAR-like element (809–1009), and a third sequence (1310–1503) containing an AG-rich tract (1310–1346; 37 bases, 78.4% AG). We propose a model suggesting the sequence requirement of the gene amplification and the replication initiation (Figure 8). We will discuss along with this model in below.

In light of the results of previous studies [20-22], these data suggest that gene amplification and replication initiation at a chromosomal locus require overlapping but not identical sequences. Replication-initiation activity requires RepP-1 and RepP-2. RepP-1 includes the AT-rich region and the MAR-like element required for gene amplification. However, gene amplification does not require RepP-2 (Figure 2), but instead requires another sequence (1310–1503; Figures 5, 6) that contains an AG-rich tract (1310–1346; 37 bases, 78.4% AG), which is a portion of a longer tract (1298–1346; 49 bases, 79.6% AG). On the other hand, Rep-P2 also contains an asymmetric AG-rich tract (45 bases, 77.8% AG). Mutations in such AG-rich tracts abrogate both replicator activity in a chromosomal context (M.I. Aladjem, personal communication) and binding of specific proteins [22]. Thus, the third region (1310–1503) required for gene amplification might be a counterpart of RepP-2. If so, both gene amplification and replication initiation might require three elements, i.e., an AT-rich region, a MAR-like element, and an AG-rich region that binds certain protein. In that case, the difference between gene amplification and replication initiation might be the choice of the AG-rich tracts. The Rep-P replicator contains another long AG-rich tract (1541–1615; 75 bases, 80.0% AG). On the other hand, the sequences within the *DHFR* IR and c-myc IR that were required for amplification contained at least two (55 bases, 80.0%; 45 bases, 75.6%) and one (68 bases, 73.5%) AG-rich tracts, respectively (Figure 7 [18]).

The differential requirement for the AG-rich tracts between replicator and HSR-generation activity might reflect the fact that the former activity was measured in a chromosomal context [20-22], whereas the latter activity might reflect extrachromosomal replication/amplification (Figures 1, 5). Several lines of evidence illustrate the differences between these two contexts. For example, a given sequence is replicated in early S phase when amplified at extrachromosomal DMs, but is replicated in late S phase when amplified at chromosomal HSRs [24]. Furthermore, a given sequence is expressed at higher levels from DMs than from HSRs [9]. DNA damage on DMs causes them to aggregate, resulting in a delay in repair [5]. Therefore, extrachromosomal localization might explain why replication initiation and chromosomal localization have different requirements for AG-rich tracts.

The *DHFR* IR and human lamin B replication origins also contain AG-rich tracts, but they could not functionally complement the AG tract in RepP-2 of ß-globin [20]. Because these origins replicate at different times, the authors of that study postulated that the AG-rich tract plays a locus-specific role that dictates replication at the correct time during S-phase. A recent report clearly showed that the replication pattern inside the immunoglobulin heavy-chain locus varies during B-cell development, and that these dynamics change as a function of transcriptional activity [25]. Thus, the choice of AG-rich tracts might explain the origin of diversification.

AT-rich tracts are commonly observed among replicators from both single-cell eukaryotes and metazoans. The AT-rich tracts in hamster *DHFR* [26,27], human c-myc [28], and human lamin B2 IRs [29] are essential for replication initiation at ectopic chromosomal sites. The AT-rich tracts are also necessary for gene amplification, not only for *DHFR* and *c-myc* IRs [18], which are constitutively early origins, but also for ß-globin IR (this study), which replicates at various times.

Many studies have demonstrated that replication initiation requires attachment to the nuclear matrix (for a recent review, see 3). Therefore, we noted in Figure 8 a MAR at the chromosomal flanking region for the replication initiation. Our data showed that amplification requires both the AR1 MAR in the vector plasmid and the MAR-like element predicted by MAR-Wiz program (Figure 7). The AR1 sequence exhibits strong *in vitro* nuclear matrix binding activity [15], thus it is an actual MAR. A fragment of the *DHFR* IR also exhibits such *in vitro* MAR activity, whereas the c-myc IR does not [1]. Such actual MAR activity has been observed in the ß-globin locus outside the Rep-P region [30-32]. Consistent with that, the MAR-Wiz program yielded the highest MAR score for the *DHFR* IR relative to the c-myc IR or ß-globin Rep-P (Figure 7B to D). We called the sequences showing moderately higher MAR-Wiz scores in the c-myc IR or ß-globin Rep-P as a MAR-like element. The element might be involved in a process other than binding to the nuclear matrix, because it actually did not bind to the nuclear matrix in vitro. The actual MAR, which binds to the nuclear matrix in vitro, is involved in the vector used in this study, and it is also found at the ß-globin locus outside the RepP-1 region. Thus, such actual MAR is needed for both replicator and gene amplification. The actual MAR is located apart from other three modules, the MAR-like, the AT-rish and the AG-rich, however these four modules may be excised together from the chromosome arm in a cell. Such excised circular molecule will be amplified and selected if it contains some additional gene that may confer growth advantage on the cell (Figure 8).

Taken together, our data reveal that the MAR element and three elements in the ß-globin Rep-P replicator, i.e., the AT-rich region, the MAR-like element, and the AG-rich region, are required for extrachromosomal amplification (Figure 8). It is important to note that these elements also appear in the minimal sequence required for HSR generation from both the *DHFR* and c-myc IRs (Figure 7 [18]). Thus, these elements are commonly required, among replicators that differ from one another with respect to replication timing and dependency on transcription, for both amplification and extrachromosomal replication.
